# Integrated convolutional neural network for skin cancer classification with hair and noise restoration

**DOI:** 10.55730/1300-0144.5954

**Published:** 2023-10-16

**Authors:** Nidhi BANSAL, Sridhar SUNDARAMURTHY

**Affiliations:** Department of Information Science and Technology, College of Engineering, Guindy, Anna University, Chennai, Tamil Nadu, India

**Keywords:** Dermoscopic images, image hair, image noise, convolutional neural network, image restoration, classification

## Abstract

**Background/aim:**

Skin lesions are commonly diagnosed and classified using dermoscopic images. There are many artifacts visible in dermoscopic images, including hair strands, noise, bubbles, blood vessels, poor illumination, and moles. These artifacts can obscure crucial information about lesions, which limits the ability to diagnose lesions automatically. This study investigated how hair and noise artifacts in lesion images affect classifier performance and how they can be removed to improve diagnostic accuracy.

**Materials and methods:**

A synthetic dataset created using hair simulation and noise simulation was used in conjunction with the HAM10000 benchmark dataset. Moreover, integrated convolutional neural networks (CNNs) were proposed for removing hair artifacts using hair inpainting and classification of refined dehaired images, called integrated hair removal (IHR), and for removing noise artifacts using nonlocal mean denoising and classification of refined denoised images, called integrated noise removal (INR).

**Results:**

Five deep learning models were used for the classification: ResNet50, DenseNet121, ResNet152, VGG16, and VGG19. The proposed IHR-DenseNet121, IHR-ResNet50, and IHR-ResNet152 achieved 2.3%, 1.78%, and 1.89% higher accuracy than DenseNet121, ResNet50, and ResNet152, respectively, in removing hairs. The proposed INR-DenseNet121, INR-ResNet50, and INR-VGG19 achieved 1.41%, 2.39%, and 18.4% higher accuracy than DenseNet121, ResNet50, and VGG19, respectively, in removing noise.

**Conclusion:**

A significant proportion of pixels within lesion areas are influenced by hair and noise, resulting in reduced classification accuracy. However, the proposed CNNs based on IHR and INR exhibit notably improved performance when restoring pixels affected by hair and noise. The performance outcomes of this proposed approach surpass those of existing methods.

## 1. Introduction

Skin cancer is the most prevalent type of cancer, accounting for millions of deaths annually worldwide. Melanoma is the deadliest form of skin cancer, causing 10,000 annual deaths worldwide [1]. Melanoma incidence increased rapidly around the world in the last 50 years [2]. The survival rate is over 95% if it is detected early but only about 15% for late detection [3]. This huge difference emphasizes the importance of melanoma detection and diagnosis at an early stage because the disease is treatable at that time. Timely detection helps in reducing mortality rates and hence saves patients’ lives. Dermoscopy is an imaging procedure that aids in the analysis of skin lesions [4]. The subsurface structures of the skin can be visually enhanced, exposing deeper skin lesions [5] and providing higher accuracy than naked-eye assessments. However, manual diagnosis requires an expert dermatologist and is also influenced by subjective variation and clinical experience, lowering the patient’s life expectancy [6]. As a result, computer-aided diagnosis (CAD) systems have emerged to help improve the efficiency of dermoscopic image analysis [7]. An accurate automatic melanoma diagnostic system is critical for assisting dermatologists in making precise diagnosis decisions and reducing the numbers of unnecessary biopsies. In the field of clinical medicine, deep neural networks have made major progress and achieved excellent results in image segmentation and classification tasks [8]. However, accurate recognition of skin lesions from dermoscopic images is challenging owing to the presence of various artifacts including hair strands, noise, air bubbles, blood vessels, clinical marks, and uneven lighting. Skin lesions may be partly obscured or covered by these artifacts, creating a partial occlusion. This kind of image with partially obscured regions makes the diagnosis of a diseased area extremely difficult [9].

Many classical techniques have been used in the literature for hair and noise removal in dermoscopic images [10–18]. Lee et al. [10] presented the first method to remove thick hairs, called Dull Razor, and applied bilinear interpolation. The PDE-based continuous morphological filter was used by Chung et al. [11] to remove undesirable hairs. Curvilinear analysis was used by Zhou et al. [12] to achieve automatic hair and ruler marking recognition, and artifacts were replaced with a feature-guided exemplar-based inpainting technique. To eliminate features from dark hair, Silveira et al. [13] introduced the morphological closing and median filter. Top-hat filtering was applied by Xie et al. [14] to eradicate thin and curled hairs followed by PDE-based inpainting. Abbas et al. [15] introduced a hair detection and repairing algorithm using a derivative of the Gaussian method to remove hair and then apply inpainting with a fast-marching method. Toossi et al. [16] implemented a canny edge detector and morphological operators to segment hairs and ruler markings. Multiresolution transport inpainting was applied to repair hair. Abuzaghleh et al. [17] proposed 84 directional filters to identify and disregard hair in images of skin lesions. Kasmi et al. presented a new method using 11 × 11 median filters to remove thin hairs and a Gabor filter for thick hairs [18]. There are various existing methods for removing noise from images [19–25]. A new method for Gaussian noise removal was proposed using multiscale filter banks [20]. A novel effective noise estimation method was proposed based on singular values of corrupted images [21].

Some deep learning methods are available for hair removal and image denoising tasks [26–30]. A convolutional neural network (CNN) was built with a postprocessing step using the Savitzky–Golay filter and Fourier domain filtering [26]. This method can detect borders belonging to hair follicles with an average Dice score of 0.83 ± 0.06. The FCN8-ResNetC-based approach for hair removal and segmentation in dermoscopic images was proposed and the obtained training accuracy was 89.38% for hair removal [27]. Jain et al. [28] proposed a fully convolutional CNN for image denoising. An image denoising and blind inpainting method was proposed to combine sparse coding with pretrained CNNs, achieving decent results for both tasks [29]. Mao et al. proposed an encoding–decoding framework for image denoising and superresolution. Their method combined convolution and deconvolution layers symmetrically by skip connections, which improved the network’s performance [30].

There are several limitations of these previous studies. First, the research available in the present body of literature generally measured hair detection accuracy and error, oblivious to the impact on skin lesion patterns. Second, despite the existence of various methods for hair and noise removal, none of the research performed to date has focused on the impact of eliminating these artifacts on the overall performance of a CAD system. It is essential to address the effects of hair lines and image noise on the classification accuracy of dermoscopic images to achieve better results and more successful treatment. In the present study, a deep learning model was developed for the removal of these artifacts. This model could be built into a complete CAD system for dermoscopic images. This study also addresses how hair and noise data affect the automatic detection of skin lesions overall. The deep learning model is run with the hair and noise artifacts and the results are compared with ground truth images. An integrated CNN with image inpainting is proposed to address unwanted hairs and restore the color and texture of skin pixels below them via dehairing with an approach referred to as integrated hair removal (IHR). This network initially performs image inpainting to eliminate unwanted hair and then integrates with deep learning models to perform classification and provide insight into the effect of removing hair. Secondly, an integrated CNN with image denoising is implemented to remove noise from images (i.e. denoising), referred to here as integrated noise removal (INR). The integrated CNN first performs image denoising to reduce noise and then integrates with the deep learning models to perform classification and evaluate the effect of removing noise. The training and validation results obtained after dehairing and denoising are compared with ground truth images. The results show that the training and validation accuracies improve when hair strands and noise are eliminated. The removal of these artifacts helps achieve better pattern analysis of dermoscopic images by deoccluding the lesion boundary or texture, resulting in more accurate classification. The core contributions of this work are as follows:

- The effects of image distortions like hair and noise on the performance of a skin CAD system are evaluated.
- Two datasets are created wherein new hairs and noise are added.
- Integrated CNNs, namely IHR and INR, are developed to leverage the advantage of removing hair and noise artifacts, being integrated with deep learning models for the improved classification of skin lesions.
- An evaluation of the performance of the proposed integrated deep learning models against the hairy and noisy datasets is conducted with extensive experimentation.
- The improved results based on accuracy and loss function when these distortions are removed are assessed.

The remainder of the paper is structured as follows: Section 2 addresses the datasets used, the proposed methodology and architecture, and network training. Section 3 presents the implementation process and experimental results. In Section 4, the results are discussed for an analysis of the performance of the proposed approach. Section 5 presents the conclusion and future research directions.

## 2. Materials and methods

### 2.1. Dataset description

The benchmark dataset HAM10000 [31] was utilized in this work. It is the International Symposium on Biomedical Imaging (ISBI) Challenge dataset available as International Skin Imaging Collaboration (ISIC) 2018, constituting a collection of 10,015 skin lesion images divided into seven categories. The seven categories are melanocytic nevus, basal cell carcinoma, actinic keratosis, melanoma, benign keratosis, dermatofibroma, and vascular lesion.

In real-life scenarios, the major artifacts causing difficulty in image analysis are hair and noise. Though the images in the selected dataset are partially occluded by artifacts such as hair, rulers, moles, and ink markings, there are very few images involving major occlusion. The major concern in the detection and assessment of lesions is the lack of an appropriate dataset with major artifacts like hair and noise. Therefore, two synthetic datasets were generated, referred to as the Hair Dataset and Noise Dataset. Hair and noise were introduced in images to obstruct the lesion area. These datasets were created to produce partial occlusion in skin cancer images and each contain a total of 5000 images. The images in the Hair Dataset were occluded by adding hair strands. For the Noise Dataset, Gaussian noise [32] was added to create partial occlusion of the lesion area. For training, 80% of the whole data were taken and for testing 20% of the data were considered. Table 1 shows images from each dataset.

#### 2.1.1. Hair Dataset

**Figure 1:**
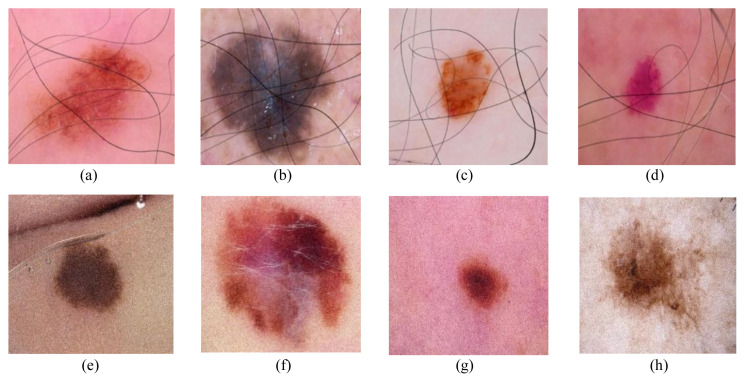
(a)–(d) present images from the Hair Dataset, while (e)–(h) present images from the Noise Dataset.

Hair creates major partial occlusion in dermoscopic images of skin. Skin images contain thick and thin hairlines. A total of 5000 images were chosen from the original HAM10000 dataset. The images chosen contained no hair or very few hairs. Hair was extracted from other dermoscopic images with more hair. This was done to maintain a natural hair artifact appearance. Hair was taken from hairy images using a masking technique and then those hairs were superimposed on selected images for the Hair Dataset. Figures 1a–1d show a few examples of Hair added to the Hair Dataset.

#### 2.1.2. Noise Dataset

A total of 5000 images were chosen from the original HAM10000 dataset and noise was added. These images were chosen from a dataset that contained no noise. Low lighting and a scarcity of resources for capturing medical images with clinical equipment result in large noise fluctuations in lesion images. Gaussian noise [32] was chosen here as it is a main source of noise in digital photos during acquisition, including sensor noise produced by inadequate lighting or transmission noise.

A typically modest amount was added or subtracted from each pixel’s original value in the image. In dermoscopic images, Gaussian noise is a major noise source arising during acquisition. All images may contain noise, varying in intensity. Here, Gaussian noise was added with zero mean and the scale (σ) varied from 1 to 30. Figures 1e–1h show a few examples of Gaussian noise added to the Noise Dataset.

### 2.2. Proposed methodology

The proposed integrated CNN model is described in this section. The methods employed for hair and noise restoration, i.e. IHR and INR, are presented. The deep learning models used for dermoscopic images and their classifications are discussed.

#### 2.2.1. Convolutional neural networks

CNNs contribute to image and video recognition tasks on a broad scale. There are several advantages to employing CNNs over standard neural networks, including the ability to learn spatial hierarchies of patterns. This enables CNNs to acquire increasingly complex and abstract visual concepts and analyze images with great efficiency. A vast number of images are necessary to train a new CNN model in a situation in which the entire network must be trained. In such a situation, all the network’s parameters must be learned from the ground up. This approach necessitates extremely large datasets, which are frequently unavailable for medical purposes. However, employing a standard network allows for the option of transfer learning.

Transfer learning is a technique that uses a model trained on one dataset as the basis for a model trained on another. The model that is already trained is known as the pretrained model. Typically, these models are built on ImageNet [33], a dataset of over 14 million images that can classify images into over 1000 different categories. In addition to using the same architecture as a standard network, one may also use parameters learned by the CNNs with earlier training on a different dataset. Therefore, to adjust the network for the classification of a new target dataset, there are two possible approaches. One is to replace only the final classification layer according to one’s target dataset, i.e. the network can be used to classify new dataset images. In the other approach, the parameters gained from the model’s training over a large dataset are fine-tuned through transfer learning. This allows the network’s early layers to extract highly generalizable patterns from a larger dataset, and the network’s later layers will take on the details of the new dataset for the adapted model.

**Figure 2:**
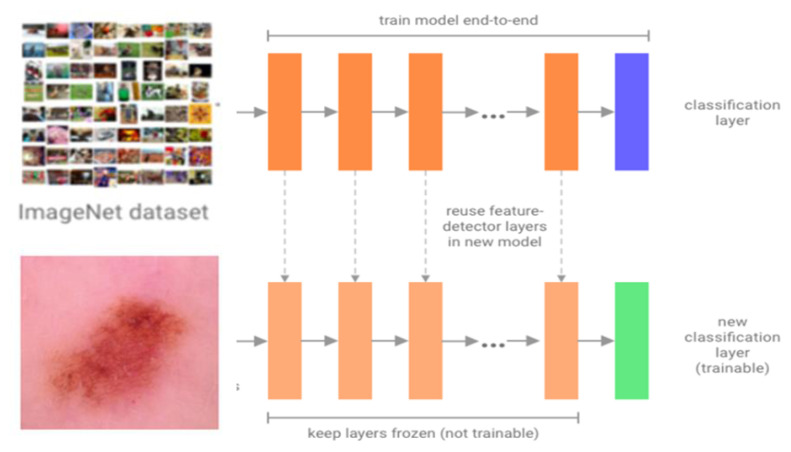
Proposed CNN for dermoscopic image classification.

In this paper, the first approach is followed: the final classification layer is modified. The proposed CNN for dermoscopic image classification is given in Figure 2. As a result, the time-consuming training stages are avoided, and benefits are gained from the features learned during the training over many images through transfer learning.

The most successful methods submitted for ISIC challenges in 2016, 2017, 2018, 2019, and 2020 used CNNs pretrained on the ImageNet database [33]. The five deep transfer learning models used in this work were ResNet50 [34], DenseNet121 [35], ResNet152 [34], VGG16 [36], and VGG19 [36]. These models were used to determine how the system performs in the event of partially occluded image data. Table 2 shows the deep learning architectures used.

#### 2.2.2. Integrated CNN with image inpainting for hair removal (dehairing)

**Figure 3:**
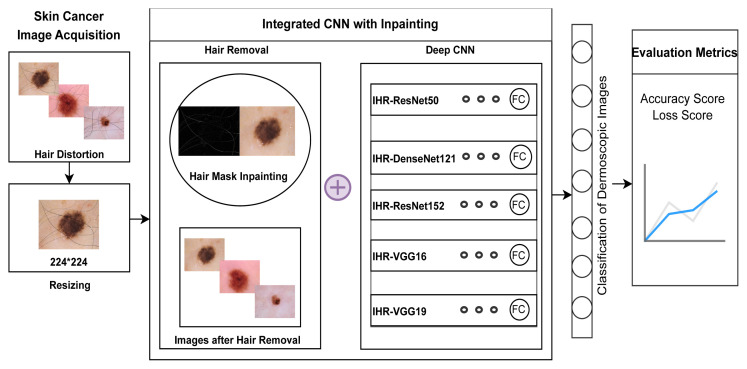
Integration of CNN with inpainting for dermoscopic hair removal and classification.

An integrated CNN with inpainting is proposed for the classification of dermoscopic images as shown in Figure 3. Integration here entails a combination of two methods: skin cancer image inpainting and classification. Inpainting is done to restore hairs by substituting them with patches that resemble the nearby pixels. This reduces the impact of hairs on diagnosis analysis. Five deep learning models were applied for the classification of refined skin cancer images. These models are referred to as IHR-ResNet50, IHR-DenseNet121, IHR-ResNet152, IHR-VGG16, and IHR-VGG19. Algorithm 1 explains an integrated CNN with inpainting for hair removal. The Hair Dataset contains 5000 images where new hairs are added, as explained in Section 2.1. The removal of dark, dense hairs and regions that resemble hair must be done properly as it aids in effective segmentation and classification of features. Numerous techniques are available in the literature for removing hair in dermoscopic images based on morphological operations [37] and thresholding [38]. Although they are fast, these techniques tend to eliminate subtle, significant features that can be mistaken for hair. An effective method for dermoscopic hair removal is the blackhat transform followed by inpainting, which is employed here as described in Algorithm 2.

The first step is to perform Gaussian blur and median blur operations before applying other methods to reduce the high-frequency data. This removes noise and edges from an image while preserving its original data. Gaussian blur is a low-pass filter that determines the variation to be applied to each pixel of the image using a Gaussian function. Its purpose is to smooth down sphere edges, which fr2uently have inconsistencies because of the marker’s rough surface. It is also used to reduce skin lines, air bubbles, light, and small hairs around the lesion. The kernel used is 3 × 3 and σ is the standard deviation of the Gaussian kernel. The median filter is a nonlinear filter and it is very effective in removing noise while preserving edges. The current pixel value is replaced with the median value in a 3 × 3 neighborhood.

The input dermoscopic image is converted from RGB to grayscale, followed by a morphological filter to find the hair contours. The morphological filter, called “blackhat,” is employed on the grayscale image. It gives the difference between the closing and the given input image. Closing eliminates the foreground’s tiny holes. The blackhat filter extracts dark objects smaller than the structuring element and finally outputs them as bright spots. An 11 × 11 cross-shaped structural element is defined. To intensify the hair contours, a thresholding operation is applied to the output of the blackhat filter. This generates a binary mask. All unrequired objects present in the dermoscopic image are discarded and only the hairlines are detected. Following this, an inpainting algorithm, TELEA [39], given in Algorithm 1, is used to restore the image by removing the hair structures from it. It preserves the appearance by replacing the hair structures with nearby pixels, producing a clear dermoscopic image. Eq. (1) shows that point *p* is inpainted as a function of all points *q* in *B_ɛ_ (p)* by summing the estimates of all points *q*, weighted by normalized weighting function *w(p, q)*:


(1) $$I(p)=\frac{\Sigma_{q\in B_{ɛ}(p)}\;w(p,q)[I(q)+\nabla I(q)(p-q)}{\Sigma_{q\in B_{ɛ}(p)}\;w(p,q)}$$

**Figure 4:**
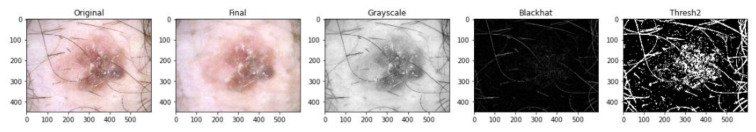
Stages of the hair removal process for dermoscopic images.

Here, *I(q)* is the original image and *I(p)* is an inpainted image. In Algorithm 3, Ω is the region to be inpainted, ∂Ω is the boundary of the region to be inpainted, and *B_ɛ_ (p)* is a neighborhood of p. To inpaint the whole Ω, we apply Eq. (1) iteratively to all pixels of ∂Ω, in increasing distance from ∂Ω’s initial position ∂Ωi. We then complete the boundary inside Ω until the whole region has been inpainted. Figure 4 shows the stages of the hair removal process.

#### 2.2.3. Integrated CNN with image denoising for noise removal (denoising)

**Figure 5:**
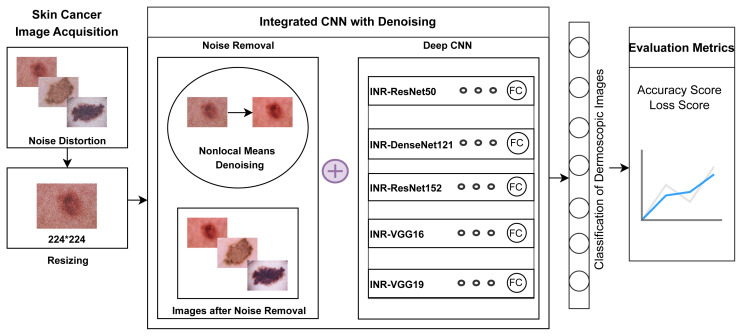
Integration of CNN with denoising for dermoscopic noise removal and classification.

An integrated CNN with noise removal is proposed for the classification of dermoscopic images as shown in Figure 5. Integration here entails a combination of two methods: skin cancer images’ noise removal and classification. Denoising is done to remove undesirable noise from images so that they can be better analyzed. Five deep learning models were applied for the classification of refined skin cancer images. These models are referred to as INR-ResNet50, INR-DenseNet121, INR-ResNet152, INR-VGG16, and INR-VGG19. Algorithm 4 presents an integrated CNN with denoising for noise removal. A total of 5000 images from the Noise Dataset, to which noise was added, are thus denoised. The process of reconstructing a signal from noisy images is referred to as denoising an image. The nonlocal (NL) means method [40] was utilized as the method for denoising to remove any probable aberrations from the images. The NL means algorithm selects a pixel, draws a small window around it, and searches the image for other windows of the same size. It then takes an average of all the windows and calculates the resultant value for the pixel. NL signifies the whole image search, not an individual region. Given a noisy image *v =* {*v (i) | i* ∈ *I*}, the *NL*[*v*] (*i*), for pixel *i*, is computed as a weighted average of all the pixels in the image, as given in Eq. (2):


(2) $$NL[v]i=\sum_{j\in I}w(i,j)v(j)$$

Here, {*w*(*i*, *j*)}*_j_* depends on the similarity between pixels *i* and *j*. It is used as the OpenCV function ‘fastNlMeansDenoisingColored.’ The function converts the image to the CIELAB color space and then separately denoises the *L* and *AB* components with given *h* parameters using the ‘FastNon-LocalMeansDenoising’ function. Larger search windows require longer denoising times. The ideal value for the luminance and color components is 10, and the higher the value, the smoother the image will be. All images from the Noise Dataset were run through this process for reconstruction.

### 2.3. Model training

Transfer learning was employed for training the IHR and INR models on the dataset, utilizing pretrained weights obtained from training on the ImageNet dataset. Five pretrained models were implemented for the given dataset. The model’s weights were loaded and the final fully connected layer was removed. The remaining part of the model was used as a feature extractor for the given dataset. A new final fully connected layer was added to get the skin lesion classes required for the output, which was 7.

The network was trained for 25 epochs. Table 1 shows the hyperparameters used to train the model. The input image for the model was a 224 × 224 × 3 RGB image. The ReLU activation function [41] was employed throughout the architecture and the optimization function used was Adam [42]. The loss function applied was categorical cross-entropy [43]. Table 3 shows all the hyperparameters and their values.

#### 2.3.1. Fully connected layer

- It is necessary to categorize the data into several classes after feature extraction, which can be achieved with a fully connected layer.
- The fully connected layer in the convolutional network takes the outcome of the convolution/pooling process and makes a classification judgement.
- *Fully connected input*: The output of the final pooling/convolutional layer is flattened, turned into a single vector, and sent as the input to the fully connected layer.
- *Fully connected output*: This gives the final probabilities for each label.
- The final layer employs the softmax activation function to determine the likelihood that the input belongs to one of several classes (classification). The class probabilities are calculated and output in a 3D array with [1 × 1 × N] dimensions, where N is the number of classes.

#### 2.3.2. ReLU activation function

- The ReLU activation function [41] is a nonlinear function that can learn complex relationships from the training data.
- ReLU is very easy to compute and implement since it only requires a comparison between its input and the value 0.
- A ReLU function will apply a max (0, x) function. The function outputs the input directly if it is positive; otherwise, it will output zero.
- The derivative remains constant, i.e. 1, for positive input and thus reduces the time taken for the model to learn and minimize the errors.
- ReLU has a predictable gradient for the backpropagation of the error. As a consequence, the computation speed is very fast.

#### 2.3.3. Categorical cross-entropy loss

- The network’s performance is measured using a metric (loss function) that counts the similarity between predicted and actual values. Cross-entropy loss is the most important cost function used in multiclass classification.
- The objective of the loss function is to optimize the model during training [43]. To optimize the loss function, parameters are modified iteratively and help in achieving correct prediction.
- The model performs better when loss is low.

## 3. Results

The proposed architecture was implemented in Google Colab. The classification accuracy and loss of the trained CNN models were calculated for training and validation. The ResNet50 [34], DenseNet121 [35], ResNet152 [34], VGG16 [36], and VGG19 [36] models were run on ground truth images from HAM10000 and corresponding images changed by the Hair Dataset and Noise Dataset. The models IHR-ResNet50, IHR-DenseNet121, IHR-ResNet152, IHR-VGG16, and IHR-VGG19 were run on the Hair Dataset after dehairing. The models INR-ResNet50, INR-DenseNet121, INR-ResNet152, INR-VGG16, and INR-VGG19 were run on the Noise Dataset after denoising the images. All models were run for 25 epochs. Results were provided for data obtained after 10, 15, and 25 epochs. The performance metrics used to validate the results were training accuracy (TAcc), training loss (TLoss), validation accuracy (VAcc), and validation loss (VLoss).

### 3.1. Experimental results on the HAM dataset

Skin cancer images were taken from the ground truth (GT) dataset (HAM). This dataset comprises 10,015 images. All models were run on these images. Table 4 shows the training and validation accuracies on the GT dataset. Table 5 shows training and validation loss on the GT dataset.

### 3.2. Experimental results with the Hair Dataset

The model performance for the Hair Dataset is shown in Tables 6–9. Table 6 shows training and validation accuracy on hair-occluded images. Table 7 shows training and validation loss on hair-occluded images. DenseNet121 gave training accuracy of 95.20% with validation accuracy of 87.10%. VGG19 with occluded hair gave training accuracy of 85.03% and validation accuracy of 78.62%.

#### 3.2.1. Dehairing results using the proposed integrated CNN with hair inpainting

Dehairing was performed using Algorithm 3 as proposed in subsection 2.2. Table 8 shows the training and validation accuracy after dehairing. Table 9 shows the training and validation loss after dehairing. It can be seen that training and validation accuracy decreased when skin images were occluded with hair strands. DenseNet121 gave training accuracy of 95.20% with hair while IHR-DenseNet121 provided 97.50% accuracy with hair removal. The validation accuracy with DenseNet121 was 87.10% when hair was present while it was 89.16% with IHR-DenseNet121 when hairs were removed. There was improvement of approximately 2% accuracy with IHR-DenseNet121. For each model, there was an increase in training and validation loss when the lesion was obstructed with hair.

**Figure 6:**
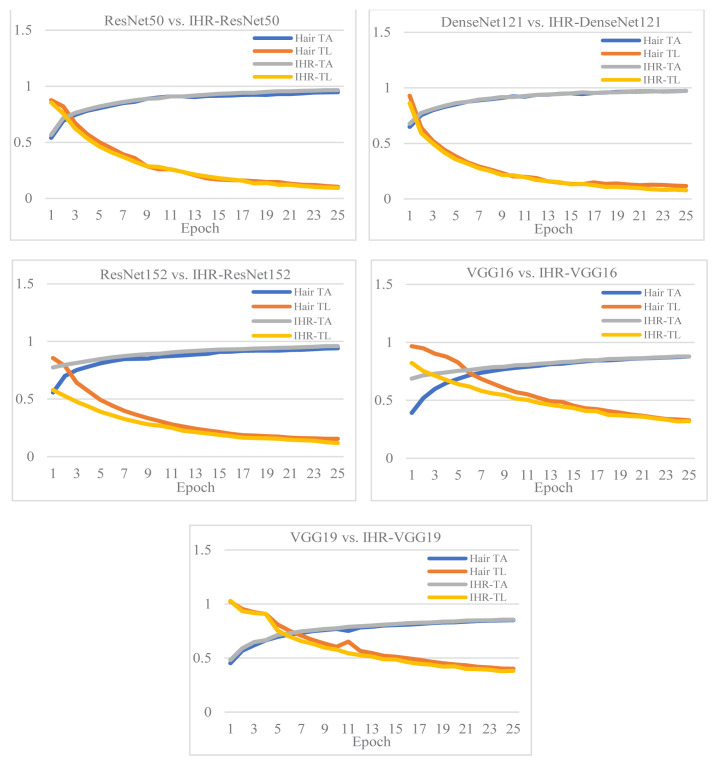
Comparison of improvement in training accuracy and loss after dehairing.

Figure 6 shows a comparison of improvement in training accuracy and loss after dehairing.

Training accuracy and loss curves were drawn and contrasted for the datasets with and without hair. It was seen that the accuracy and loss curves after dehairing with the proposed IHR models were better and show improved results compared to those obtained with hair.

### 3.3. Experimental results with Noise Dataset

The model performance for the Noise Dataset is shown in Tables 10–13. Table 10 shows the training and validation accuracy obtained for noise-occluded images. Table 11 shows the training and validation loss for noise-occluded images. DenseNet121 achieved the highest training accuracy of 96.04% and validation accuracy of 86.50%. VGG19 with occluded noise gave training accuracy of 78.25% and validation accuracy of 76.75%.

#### 3.3.1. Denoising results using the proposed integrated CNN with nonlocal means denoising

Denoising was performed using Algorithm 4 as proposed in subsection 2.2. Table 12 shows the training and validation accuracy after denoising. Table 13 shows the training and validation loss after denoising. It can be seen that the training and validation accuracy decreased when skin images were distorted with noise. DenseNet121 gave training accuracy of 96.04% with noise, while a rate of 97.45% was achieved with INR-DenseNet121 when the noise was removed.

The validation accuracy with DenseNet121 was 86.50% when noise was present, while INR-DenseNet121 gave 87.58% accuracy when the noise was removed. There was improvement of approximately 1% in accuracy with INR-DenseNet121. For each model, there was an increase in training and validation loss when the lesions were obstructed with noise.

**Figure 7:**
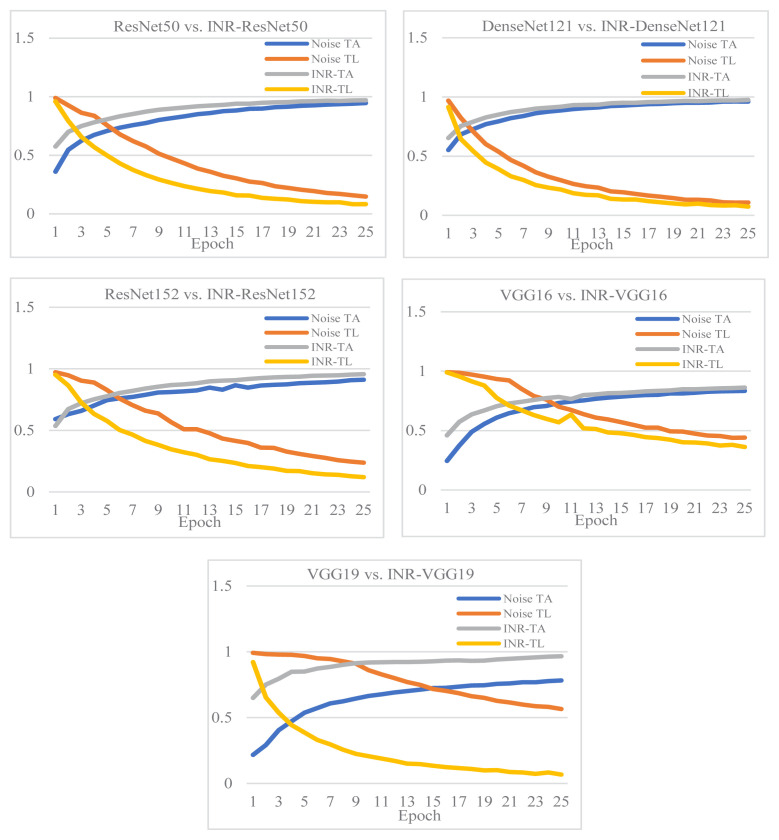
Comparison of improvement in training accuracy and loss after denoising.

Figure 7 shows a comparison of the improvement in training accuracy and loss after denoising. The training accuracy and loss curves were drawn and contrasted for the datasets with and without noise. It was seen that the INR models gave more accurate output. The accuracy and loss curves after denoising were better and showed improved results compared to the results obtained with noise.

### 3.4. Comparison of ground truth with hair, noise, dehairing, and denoising

**Figure 8:**
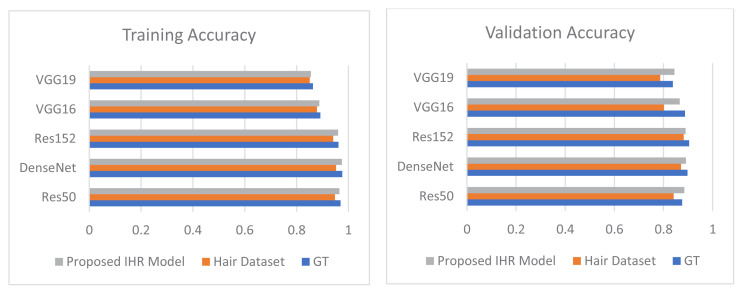
Comparison of improvement in training and validation accuracy for GT, Hair Dataset, and proposed IHR model.

**Figure 9:**
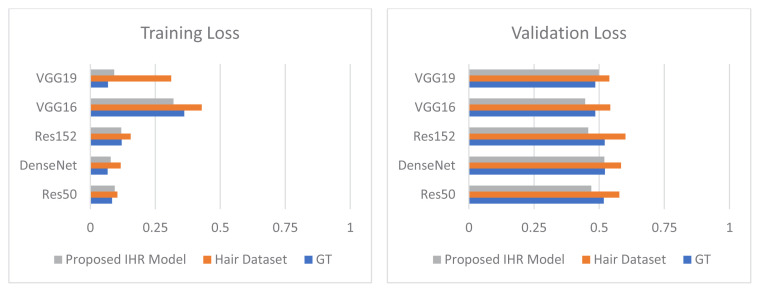
Comparison of improvement in training and validation loss for GT, Hair Dataset, and proposed IHR model.

Extensive experimentation was performed to analyze the effect of distortions on the overall diagnosis of skin lesions. Comparison graphs were drawn to compare ground truth results with the occluded datasets (Hair Dataset and Noise Dataset) and the refined datasets with dehairing and denoising. Figure 8 shows a comparison between the training and validation accuracy rates for the ground truth, hairy images, and dehaired images. Figure 9 shows a comparison between the training and validation loss for the ground truth, hairy images, and dehaired images.

**Figure 10:**
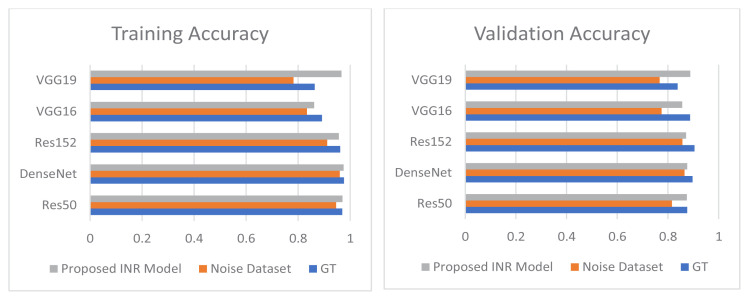
Comparison of improvement in training and validation accuracy for GT, Noise Dataset, and proposed INR model.

**Figure 11:**
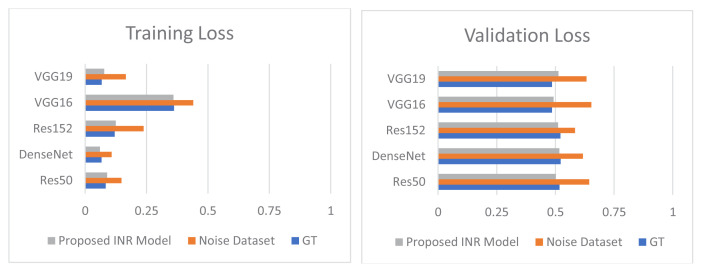
Comparison of improvement in training and validation loss for GT, Noise Dataset, and proposed INR model.

The proposed IHR model was employed for dehairing and the proposed INR model was employed for denoising. Figure 10 shows a comparison between the training and validation accuracy rates for the ground truth, noised images, and denoised images. Figure 11 shows a comparison between training and validation loss for the ground truth, noised images, and denoised images.

From Figures 10–13, it can be seen that the proposed IHR-DenseNet121 achieved 2.3% higher accuracy than DenseNet121 with hair occlusion and the proposed INR-DenseNet121 achieved 1.41% higher accuracy than DenseNet121 with noise occlusion.

The appearances often resulted in low accuracy and high loss in skin lesion classification. The comparison of the results obtained by deep learning models with and without artifacts revealed a significant difference in employing a method for restoring the distorted parts.

## 4. Discussion

The performance of the proposed hair removal method was compared with published hair detection and segmentation algorithms. Table 14 shows the accuracy metric computed for all algorithms in the presence of hair and after dehairing. The proposed noise removal was also compared with available methods for denoising. Table 15 shows the accuracy metric computed for all algorithms after denoising.

Tables 14 and 15 show that the proposed methods achieved results comparable to those of existing computer vision techniques. The proposed IHR-DenseNet121, IHR-ResNet50, and IHR-ResNet152 outperformed the existing methods for dehairing. The INR-DenseNet121, INR-ResNet50, and INR-VGG19 models for noise removal were also superior to the available methods in the literature. The proposed methods can remove elements causing partial occlusion with more accuracy and allow precise classification of lesions according to class.

In this work, the effects of skin images occluded with hair and noise were analyzed. Components such as hair and noise affect image quality and cause classification inaccuracies. These artifacts disrupt the features that are occluded behind them. If a lesion feature is not accurately determined, the diagnosis may not be correct. Therefore, it is necessary to diminish the effect of such elements.

This is the first work in which 5000 images were adulterated with hair and noise. The projected model can successfully eliminate the effects of occluded regions, thereby resulting in better precision. Skin lesions bounded by undesirable artifacts of hair and noise were successfully corrected and classified with the inclusion of the IHR and INR models with inpainting and NL means, respectively. These methods mask any hair or noise hiding the lesion and preserve the features occluded by them. The examination of results after applying these methods showed that the integrated models are capable of effectively classifying skin lesions regardless of the presence of unwanted artifacts. This automatic and efficient CAD system can help in the robust analysis of skin lesions in dermoscopic images, saving time for both doctors and patients.

## 5. Conclusion

Skin lesion images suffer from artifacts like hairy pixels, noise, poor color contrast, low illumination, moles, bubbles, and resolution. In this work, datasets were created with hair and noise to make a CAD system applicable for more realistic scenarios. The hair strands in skin lesion images add extra features that can lead to misdiagnosis. Noise artifacts diminish the visual quality of digital images, lowering the precision and accuracy of image analysis operations. The effect of noise and hair artifacts on diagnostic accuracy was studied here and it was found that these artifacts lack accuracy and can be a reason for inaccurate analysis. Noise and hair removal techniques can enhance image quality. Removal and restoration of the regions after hair and noise removal is vital so that features within lesions can be examined more thoroughly and quickly. The proposed integrated CNNs with IHR and INR allow for improved and accurate diagnosis of lesions from dermoscopic images after image restoration. This analysis is crucial in studying unwanted segmentation and classification results of lesion images due to the presence of the hairs and noise covering them. The output of the proposed methods allows more accurate and high-quality results. Many other artifacts like ruler marks, color charts, ink marks, moles, fuzzy borders, or numerous shades of color should also be isolated and corrected. There is a need for an automatic hair removal method that preserves all lesion features in the presence of all of these artifacts while keeping its computational cost low for accurate melanoma recognition and classification tasks. Future work will focus on developing a deep learning method for image inpainting and restoration.

## Figures and Tables

**Table 1:** Number of images per category in the dataset.

Dataset	Description	Number of images opted for occlusion	Number of images for classification
Dataset 1	Original ground truth	-	10,015
Dataset 2	Hair strands	5000	10,015
Dataset 3	Noise (Gaussian)	5000	10,015

**Table 2:** Details of deep learning architectures.

Features	ResNet50	DenseNet121	ResNet152	VGG16	VGG19
Number of Layers	50	121	152	16	19
Top five accuracy	0.921	0.923	0.931	0.901	0.900
Number of parameters	25 million	8 million	60 million	138 million	143 million
Size	98 MB	33 MB	232 MB	528 MB	549 MB
Depth	168	121	-	23	26

**Table 3:** Hyperparameters for the proposed work.

Serial number	Name of hyperparameter	Value of hyperparameter
1.	Input size	224 × 224 × 3
2.	Batch size	32
3.	Epochs	25
4.	Optimization function	Adam
5.	Learning rate	1e-3
6.	Loss function	Categorical cross-entropy
7.	Activation function	ReLU

**Table 4:** Training and validation accuracy on the GT dataset.

Epoch	ResNet50		DenseNet121		ResNet152		VGG16		VGG19	
	TAcc	VAcc	TAcc	VAcc	TAcc	VAcc	TAcc	VAcc	TAcc	VAcc
10	0.9027	0.8407	0.9382	0.8701	0.8921	0.8595	0.8132	0.8210	0.7827	0.8129
15	0.9414	0.8803	0.9569	0.8970	0.9255	0.9004	0.8520	0.8603	0.8230	0.8166
25	0.9694	0.8756	0.9763	0.8972	0.9614	0.9043	0.8918	0.8872	0.8634	0.8380

**Table 5:** Training and validation loss on the GT dataset.

Epoch	ResNet50		DenseNet121		ResNet152		VGG16		VGG19	
	TLoss	VLoss	TLoss	VLoss	TLoss	VLoss	TLoss	VLoss	TLoss	VLoss
10	0.2631	0.4683	0.1663	0.4030	0.3471	0.4254	0.5697	0.5685	0.2079	0.5010
15	0.159	0.4446	0.1167	0.5547	0.2351	0.5132	0.4771	0.4619	0.1345	0.4619
25	0.0832	0.5175	0.0665	0.5219	0.1202	0.5213	0.3617	0.4852	0.0675	0.4852

**Table 6:** Training and validation accuracy on the Hair Dataset.

Epoch	ResNet50		DenseNet121		ResNet152		VGG16		VGG19	
	TAcc	VAcc	TAcc	VAcc	TAcc	VAcc	TAcc	VAcc	TAcc	VAcc
10	0.9001	0.7932	0.9131	0.8283	0.8688	0.8423	0.7811	0.7731	0.7693	0.7273
15	0.9264	0.8129	0.9398	0.8447	0.9096	0.8752	0.8275	0.7922	0.8053	0.7710
25	0.9478	0.8411	0.952	0.8710	0.9412	0.8819	0.8780	0.8018	0.8503	0.7862

**Table 7:** Training and validation loss on the Hair Dataset.

Epoch	ResNet50		DenseNet121		ResNet152		VGG16		VGG19	
	TLoss	VLoss	TLoss	VLoss	TLoss	VLoss	TLoss	VLoss	TLoss	VLoss
10	0.2609	0.4465	0.204	0.4081	0.3087	0.4290	0.5705	0.5050	0.6049	0.5174
15	0.1688	0.4651	0.1352	0.5044	0.2144	0.4828	0.4538	0.4505	0.513	0.5715
25	0.1038	0.5772	0.1165	0.5837	0.1553	0.6012	0.4287	0.5424	0.3114	0.5390

**Table 8:** Training and validation accuracy after dehairing on the Hair Dataset.

Epoch	IHR-ResNet50		IHR-DenseNet121		IHR-ResNet152		IHR-VGG16		IHR-VGG19	
	TAcc	VAcc	TAcc	VAcc	TAcc	VAcc	TAcc	VAcc	TAcc	VAcc
10	0.8918	0.8506	0.9184	0.8556	0.8949	0.8481	0.8134	0.7814	0.7754	0.8534
15	0.9327	0.8738	0.9506	0.8836	0.9288	0.8524	0.8549	0.8179	0.8142	0.8229
25	0.9656	0.8847	0.9750	0.8916	0.9601	0.89	0.8879	0.8663	0.8546	0.8445

**Table 9:** Training and validation loss after dehairing on the Hair Dataset.

Epoch	IHR-ResNet50		IHR-DenseNet121		IHR-ResNet152		IHR-VGG16		IHR-VGG19	
	TLoss	VLoss	TLoss	VLoss	TLoss	VLoss	TLoss	VLoss	TLoss	VLoss
10	0.2819	0.4567	0.2122	0.3898	0.2701	0.4991	0.5144	0.5511	0.2788	0.4354
15	0.1810	0.4651	0.1318	0.4114	0.1890	0.4860	0.433	0.5582	0.1861	0.4567
25	0.0938	0.4696	0.0779	0.5198	0.1187	0.4581	0.3202	0.4461	0.0914	0.4985

**Table 10:** Training and validation accuracy on the Noise Dataset.

Epoch	ResNet50		DenseNet121		ResNet152		VGG16		VGG19	
	TAcc	VAcc	TAcc	VAcc	TAcc	VAcc	TAcc	VAcc	TAcc	VAcc
10	0.8179	0.7791	0.8871	0.8265	0.8120	0.7712	0.7322	0.7873	0.6642	0.7738
15	0.8821	0.8284	0.9275	0.8623	0.8658	0.8200	0.7848	0.7747	0.7251	0.7694
25	0.9462	0.8158	0.9604	0.8650	0.9114	0.8570	0.8339	0.7748	0.7825	0.7675

**Table 11:** Training and validation loss on the Noise Dataset.

Epoch	ResNet50		DenseNet121		ResNet152		VGG16		VGG19	
	TLoss	VLoss	TLoss	VLoss	TLoss	VLoss	TLoss	VLoss	TLoss	VLoss
10	0.4723	0.6156	0.2975	0.5243	0.5669	0.4925	0.7012	0.5876	0.2613	0.5627
15	0.3038	0.5725	0.1949	0.5104	0.4144	0.5598	0.5709	0.6058	0.2171	0.6096
25	0.1475	0.6439	0.1078	0.6173	0.2378	0.5835	0.4398	0.6532	0.1656	0.6328

**Table 12:** Training and validation accuracy after denoising on the Noise Dataset.

Epoch	INR-ResNet50		INR-DenseNet121		INR-ResNet152		INR-VGG16		INR-VGG19	
	TAcc	VAcc	TAcc	VAcc	TAcc	VAcc	TAcc	VAcc	TAcc	VAcc
10	0.8982	0.8525	0.9179	0.8883	0.8672	0.8470	0.7824	0.7744	0.9194	0.8695
15	0.9404	0.8713	0.9505	0.8453	0.9080	0.8425	0.8169	0.8373	0.9272	0.8731
25	0.9701	0.8747	0.9745	0.8758	0.9565	0.8710	0.8617	0.8560	0.9665	0.8882

**Table 13:** Training and validation loss after denoising on the Noise Dataset.

Epoch	INR-ResNet50		INR-DenseNet121		INR-ResNet152		INR-VGG16		INR-VGG19	
	TLoss	VLoss	TLoss	VLoss	TLoss	VLoss	TLoss	VLoss	TLoss	VLoss
10	0.2631	0.4683	0.2178	0.4030	0.3471	0.4254	0.5697	0.5685	0.2079	0.5010
15	0.159	0.4446	0.1338	0.5547	0.2351	0.5332	0.4771	0.4619	0.1345	0.5318
25	0.0895	0.5015	0.0602	0.5167	0.1242	0.5113	0.3592	0.4922	0.0775	0.5130

**Table 14:** Comparison with existing hair removal methods.

Year	Method used	Accuracy with hair occlusion	Accuracy posthair removal
(1997) [10]	Dullrazor	-	93.15
(2011) [44]	PDE	-	91.74
(2013) [45]	Curvilinear matched filtering	-	81.13
(2013) [46]	Derivative of Gaussians	-	87.36
(2015) [47]	Threshold decomposition	-	80.13
(2017) [48]	ED + MBL	-	90.99
(2021) [49]	SharpRazor	-	93.80
Proposed IHR	Inpainting + DenseNet121	95.2	**97.50**
	Inpainting + ResNet50	94.78	**96.56**
	Inpainting + ResNet152	94.12	**96.01**

**Table 15:** Comparison with existing noise removal methods.

Year	Method used	Accuracy with noise occlusion	Accuracy postnoise removal
(2015) [50]	U-Net	-	87.25
(2021) [51]	DP-LinkNet	-	94.86
Proposed INR	Nonlocal Means + DenseNet121	96.04	**97.45**
	Nonlocal Means + ResNet 50	94.62	**97.01**
	Nonlocal Means + VGG19	78.25	**96.65**

**Algorithm 1:** Integrated CNN with Inpainting for Hair Removal

Input: Skin Images from HAM10000
Output: Hair removal Inpainted results with Accuracy and Loss
1) Input Skin cancer Images M_1_……. M_n_
2) For each Image M_i_,
do
Dehair_Inpainted (M_i_, Kernel, Mask)
3) For each Dehair_Inpainted image M_i_, resize = 224*224
4) Fine-tune the last fully connected (FC) layer of deep CNN to identify the probabilities of seven skin cancer classes.
5) Train five deep CNNs IHR-ResNet50, IHR-DenseNet121, IHR-ResNet152, IHR-VGG16 and IHR-VGG19.
6) Validate the model and calculate training and validation accuracy and loss for performance evaluation.

**Algorithm 2:** Dehair_Inpainted (Image, Kernel, Mask)

Input: Image, Kernel, Mask
Output: Skin images with Inpainted Hair
G_Blur ← GaussianBlur (Image, Kernel * Kernel, σ)
Med_blur ← MedianBlur (G_Blur, Kernel)
Image_GrayScale ← Color (Med_blur, RGB2GRAY)
Kernel1 ← StructuringElement (Morph_Cross, Kernel)
Blackhat ← MorphologyEx (Image_GrayScale, MORPH_BLACKHAT, Kernel1)
ret_v, Thresh2_Image ← Threshold (Blackhat, Thresh, Thresh_MaxVal, THRESH_BINARY)
Output_Image ← Inpaint (Med_blur, Thresh2_Image, 1, INPAINT_TELEA)
Dehair_Inpainted ← Color (Output_Image, COLOR_BGR2RGB)

**Algorithm 3:** INPAINT_TELEA

δΩi = boundary of the region to inpaint
δΩ = δΩi
while (δΩ not empty)
{
p = pixel of δΩ closest to δΩi
inpaint p using Eq.1
advance δΩ into Ω
}

**Algorithm 4:** Integrated CNN with Non-Local Means for Denoising

Input: Skin Images from HAM10000
Output: Noise removal results with Accuracy and Loss
1) Input Skin cancer Images M_1_….…. Mn
2) For each Image M_i_,
Denoise ← fastNlMeansDenoisingColored (Input_img, Out_Image,
Lum_comp, color_comp, template_win, search_win)
3) For each Denoised image M_i_, resize = 224*224
4) Fine-tune the last fully connected (FC) layer of deep CNN to identify the probabilities of skin cancer classes.
5) Train five deep CNNs INR-ResNet50, INR-DenseNet121, INR-ResNet152, INR-VGG16 and INR-VGG19.
6) Validate the model and calculate training and validation accuracy and loss for performance evaluation.
